# Benefit of second-line systemic chemotherapy for advanced biliary tract cancer: A propensity score analysis

**DOI:** 10.1038/s41598-019-42069-1

**Published:** 2019-04-03

**Authors:** Florian Moik, Jakob M. Riedl, Thomas Winder, Angelika Terbuch, Christopher H. Rossmann, Joanna Szkandera, Thomas Bauernhofer, Anne-Katrin Kasparek, Renate Schaberl-Moser, Andreas Reicher, Felix Prinz, Martin Pichler, Herbert Stöger, Michael Stotz, Armin Gerger, Florian Posch

**Affiliations:** 1 Division of Oncology, Department of Internal Medicine, Medical University of Graz, Auenbruggerplatz 15, 8036 Graz, Austria; 2 Clinical Division of Haematology & Haemostaseology, Department of Medicine I, Medical University of Vienna, Währinger Gürtel 18-20, 1090 Vienna, Austria; 3 Division of Oncology, Department of Internal Medicine II, Academic Teaching Hospital Feldkirch, Carinagasse 47, 6800 Feldkirch, Austria; 4 Research Center for Molecular Medicine (CeMM) of the Austrian Academy of Sciences, Lazarettgasse 14, 1090 Vienna, Austria; 5 Department of Experimental Therapeutics, The University of Texas MD Anderson Cancer Center, 1901 East Road, Room 3SCR4.3424, Houston, Texas 77054 USA; 6 Center for Biomarker Research in Medicine (CBmed), Stiftingtalstrasse 5, 8010 Graz, Austria

## Abstract

Whether 2^nd^-line-chemotherapy (2LCTX) + best-supportive-care (BSC) benefits patients with advanced biliary tract cancer (aBTC) more than BSC alone is unclear. We therefore conducted a propensity-score-based comparative effectiveness analysis of overall survival (OS) outcomes in 80 patients with metastatic, recurrent, or inoperable aBTC, of whom 38 (48%) were treated with BSC + 2LCTX and 42 (52%) with BSC alone. After a median follow-up of 14.8 months and 49 deaths, the crude 6-, 12-, and 18-month Kaplan-Meier OS estimates were 77%, 53% and 23% in the BSC + 2LCTX group, and 29%, 21%, and 14% in patients in the BSC group (p = 0.0003; Hazard ratio (HR) = 0.36, 95%CI:0.20–0.64, p = 0.001). An inverse-probability-of-treatment-weighted (IPTW) analysis was conducted to rigorously account for the higher prevalence of favorable prognostic variables in the 2LCTX + BSC group. After IPTW-weighting, the favorable association between 2LCTX and OS prevailed (adjusted HR = 0.40, 95%CI: 0.17–0.95, p = 0.037). IPTW-weighted 6-, 12-, and 18-month OS estimates were 77%, 58% and 33% in the BSC + 2LCTX group, and 39%, 28% and 22% in the BSC group (p = 0.037). Moreover, the benefit of 2LCTX was consistent across several clinically-relevant subgroups. Within the limitations of an observational study, these findings support the concept that 2LCTX + BSC is associated with an OS benefit over BSC alone in aBTC.

## Introduction

Advanced biliary tract cancer (aBTC) is an aggressive orphan malignancy with a dismal prognosis, and is commonly defined as carcinoma of the intra- or extrahepatic bile ducts or the gallbladder that is inoperable locally advanced, recurrent, or metastatic^1,2^. Despite recent progress in the molecular understanding of this disease, new targeted treatment approaches have not made their way to the clinic yet, and systemic chemotherapy remains the mainstay of treatment^3–5^. While first-line systemic chemotherapy (1LCTX) in palliative intent is an accepted treatment strategy for improving survival and quality-of-life in these patients^6–9^, the benefit of second-line systemic chemotherapy (2LCTX) is less clear^10^. Both best supportive care alone (BSC) and BSC + 2LCTX are practicable options in the second-line setting^11^, but considerable uncertainty exists to date about which of these two treatment approaches will benefit patients most. Observational evidence in support of the 2LCTX + BSC approach is based on retrospective cohort studies of aBTC patients treated with fluoropyrimidine-based mono- or polychemotherapy^10–18^. However, due to their single-arm design, these studies cannot address the overall benefit of 2LCTX over BSC. Importantly, a systematic comparison of outcomes in patients receiving 2LCTX + BSC versus patients treated with BSC alone is currently lacking^19,20^.

In the absence of randomized data comparing 2LCTX + BSC vs. BSC in this setting, comparative effectiveness analyses of observational data may provide some guidance for physicians on the relative efficacy of these two treatment approaches^21^. However, a non-randomized comparison of these two treatment strategies has a high risk of bias, because patients receiving 2LCTX + BSC are likely selected on the basis of favorable oncologic and comorbidity profile, whereas patients assigned to BSC alone are likely to have poor performance status, comorbidities, obstructive bile duct pathology, or profoundly progressive disease rendering them ineligible for a further course of cytotoxic chemotherapy^11^. This could lead to an overestimation of the “true” effect of 2LCTX in this setting. To address this problem, comparative effectiveness research methods have been developed^22^.

In this observational study, we aim to explore potential overall survival benefits of 2LCTX + BSC vs. BSC alone in patients with aBTC after first-line systemic chemotherapy. A propensity score analysis using inverse-probability-of-treatment-weights (IPTW) was implemented to rigorously account for non-random treatment assignment to 2LCTX.

## Results

### Baseline characteristics and crude overall survival estimates

Eighty patients were included in the analysis (Table 1). At baseline, the median age of the cohort was 68.0 years [25^th^–75^th^ percentile: 60.0–73.0], 38 patients (48%) were female, and the median Karnofsky Index was 90% [80–90]. Most patients’ tumors were moderately differentiated (tumor grade G2: n = 43 (96%)) and were adenocarcinomas (n = 78 (98%)). During first-line chemotherapy, 20 patients (25%) had received Cisplatin/Gemcitabine, 45 (56%) had received Gemcitabine monochemotherapy, and 15 patients (19%) were treated with other regimens. The objective response rate in 1^st^-line CTX was 20% (95%CI: 12–30). After baseline, patients were followed-up for a median interval of 14.8 months (25^th^–75^th^ percentile: 5.0–24.6). During this interval we observed 49 deaths (61%), of which 46 (94%) were adjudicated to aBTC. Causes-of-death not adjudicated to aBTC were cardiorespiratory arrest (n = 1), heart failure (n = 1), and acute bleeding from esophageal varices (n = 1). The 3-, 6-, 12-, and 18-month OS estimates were 69% (95%CI: 57–78), 54% (42–65), 38% (26–50), and 19% (9–31), respectively (Supplementary Fig. 1).

**Table 1:** Baseline characteristics of the study population (n = 80).

Variable	n (% miss.)	Overall (n = 80)	BSC only (n = 42)	2LCTX + BSC (n = 38)	p*	Δ_S_	Δ_S-IPTW_20_	Δ_S-IPTW_8_
**Demographics**								
Age (years)	80 (0%)	68.0 [60.9–73.0]	69.5 [63.0–73.7]	67.1 [57.5–72.3]	0.16	0.26	0.27	0.30
Female Gender	80 (0%)	38 (48%)	24 (57%)	14 (37%)	0.07	0.41	0.02	0.05
BMI (kg/m²)	70 (13%)	24.4 [22.3–26.9]	24.0 [21.8–27.1]	24.8 [22.4–26.6]	0.49	0.15	0.03	0.17
History of smoking	72 (10%)	19 (26%)	11 (31%)	8 (22%)	0.42	0.19	0.16	0.06
Charleson Comorbidity Index	80 (0%)	8 [7–10]	9 [7–10]	8 [7–9]	0.69	0.02	0.03	0.06
Synchronous aBTC	71 (12%)	36 (51%)	10 (29%)	24 (71%)	0.001	0.83	0.43	0.21
**Performance status**	60 (25%)	/	/	/	/	/	/	
Karnofksy Index	/	90 [80–90]	80 [70–90]	90 [80–90]	0.0001	1.21	0.31	0.59
–ECOG 0	/	33 (55%)	10 (34%)	23 (74%)	0.002	0.85	0.23	0.24
–ECOG 1–2	/	27 (45%)	19 (66%)	8 (26%)				
**Tumor location**	80 (0%)	/	/	/	/	/	/	
–Gallbladder	/	23 (29%)	17 (40%)	6 (16%)	0.006	0.56	0.04	0.12
–Intrahepatic	/	30 (38%)	17 (40%)	13 (34%)		0.13	0.08	0.18
–Perihilar/Klatskin	/	9 (11%)	2 (5%)	7 (18%)		0.43	0.48	0.22
–Distal/Ampulla	/	14 (18%)	3 (7%)	11 (29%)		0.58	0.43	0.18
–CUP-CCC	/	4 (5%)	3 (7%)	1 (3%)		0.21	0.72	0.40
**Tumor histology**	80 (0%)	/	/	/	/	/	/	
–Adenocarcinoma	/	78 (98%)	41 (98%)	37 (98%)	0.99	0.02	0.03	0.01
–Others	/	2 (3%)	1 (2%)	1 (3%)				
**Tumor grade**	75 (6%)	/	/	/	/	/	/	
–G1 or G2	/	46 (61%)	22 (56%)	24 (67%)	0.60	0.21	0.14	0.41
–G3	/	29 (57%)	17 (44%)	12 (33%)				
**1** ^**st**^ **line CTX data**	80 (0%)	/	/	/	/	/	/	
–Cisplatin/Gemcitabine	/	20 (25%)	10 (24%)	10 (26%)	0.47	0.06	0.28	0.44
–Gemcitabine mono	/	45 (56%)	26 (62%)	19 (50%)		0.24	0.39	0.31
–Other CTX regimens	/	15 (19%)	6 (14%)	9 (24%)		0.24	0.18	0.13
Objective response	80 (0%)	16 (20%)	8 (19%)	8 (21%)	0.82	0.05	0.09	0.21
Number of cycles	79 (1%)	4 [2–6]	3 [2–6]	4 [3–5]	0.10	0.36	0.45	0.48
**2** ^**nd**^ **line CTX data**	38 (0%)	/	/	/	/	/	/	
–Fluoropyrimidine mono	/	N/A	N/A	26 (68%)	N/A	N/A	N/A	N/A
–Fluoropyrimidine-based combinations	/	N/A	N/A	8 (21%)	N/A	N/A	N/A	N/A
–Other CTX regimens	/	N/A	N/A	4 (11%)	N/A	N/A	N/A	N/A
Objective response rate	38 (0%)	N/A	N/A	1 (3%)	N/A	N/A	N/A	N/A
Number of cycles	36 (5%)	N/A	N/A	4 [2–8]	N/A	N/A	N/A	N/A
**Laboratory parameters**	/	/	/	/	/	/	/	
Haemoglobin (g/dL)	78 (3%)	11.7 [10.1–12.6]	10.2 [9.8–12.1]	12.4 [11.3–13.1]	0.002	0.62	0.57	0.64
Leukocyte count (G/L)	78 (3%)	7.4 [5.2–9.4]	7.7 [5.7–10.4]	6.9 [5.1–9.1]	0.26	0.39	0.03	0.44
Neutrophil count (G/L)	78 (3%)	4.8 [3.0–6.4]	5.5 [3.5–8.3]	4.4 [2.8–5.4]	0.06	0.41	0.39	0.10
Lymphocyte count (G/L)	78 (3%)	1.4 [1.1–1.7]	1.3 [0.9–1.7]	1.4 [1.1–2.1]	0.12	0.47	0.30	0.17
Platelet count (G/L)	78 (3%)	225 [138–343]	225 [134–307]	224 [141–428]	0.45	0.33	0.18	0.11
C-reactive protein (mg/dL)	77 (4%)	21.6 [7.7–47.1]	32.5 [11.4–62.0]	12.6 [5.2–34.2]	0.03	0.45	0.15	0.41
Bilirubin (mg/dL)	77 (4%)	0.6 [0.4–1.3]	0.8 [0.4–2.0]	0.5 [0.4–0.9]	0.06	0.49	0.19	0.44
Gamma-GT (units/L)	77 (4%)	311 [102–577]	336 [99–613]	245 [113–394]	0.43	0.12	0.23	0.18
Alkalic Phosphatase (units/L)	77 (4%)	168 [106–352]	208 [113–378]	152 [96–268]	0.32	0.34	0.01	0.27
AST (units/L)	77 (4%)	46 [30–80]	47 [33–83]	37 [27–66]	0.12	0.38	0.30	0.39
ALT (units/L)	77 (4%)	34 [20–62]	34 [19–70]	32 [20–59]	0.74	0.16	0.04	0.07
Albumin (g/dL)	77 (4%)	3.7 [3.2–4.0]	3.4 [2.9–3.9]	3.9 [3.6–4.1]	0.0007	0.63	0.52	0.70
CEA	48 (40%)	3.6 [1.8–11.0]	4.3 [1.8–11.1]	3.4 [1.9–10.9]	0.98	0.12	0.08	0.10
CA19–9	48 (40%)	144 [21–944]	223 [14–3204]	97 [29–433]	0.56	0.21	0.14	0.34

Distribution overall and by treatment assignment to 2LCTX + BSC versus BSC. Continuous variables are summarized as medians [25^th^ percentile (Q1) – 75^th^ percentile (Q3)], whereas categorical variables are reported as absolute frequencies and percentages. *p-values for difference between 2LCTX + BSC vs. BSC alone are from Pearson’s chi-squared tests (categorical variables with expected cell counts ≥5), Fisher’s exact tests (categorical variables with expected cell counts < 5), or Wilcoxon rank-sum tests (continuous variables); Abbreviations: n (%miss.) – number of patients with fully observed data (% missing from a total of 80 patients), BSC – best supportive care, 2LCTX + BSC – 2^nd^-line chemotherapy and BSC, Δ_S_ – Standardized mean difference (SMD), Δ_S-IPTW_20_ – IPTW-weighted SMD (weighing with the main IPTW based on a 20-variable propensity score model as reported in Supplementary Table 2), Δ_S-IPTW_8_ – IPTW-weighted SMD (weighing with the “sensitivity analysis” IPTW based on an 8-variable propensity score model as reported in Supplementary Table 3), BMI – body mass index, ECOG – Eastern Cooperative Oncology Group performance status, CUP-CCC – cancer of unknown primary with cholangiocellular differentiation, CTX – chemotherapy, AST – Aspartate aminotransferase, ALT – Alanine aminotransferase, CEA – carcinoembryonic antigen, CA 19–9 – Cancer antigen 19–9.

### Crude analysis of overall survival according to 2^nd^-line treatment group

**Figure 1:**
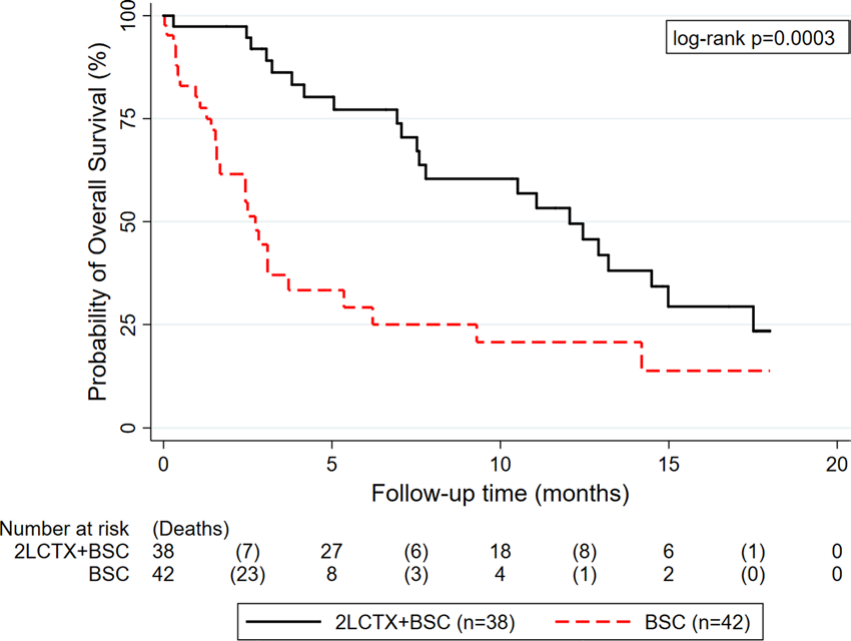
Unadjusted Kaplan-Meier curves of overall survival according to treatment assignment to 2LCTX + BSC versus BSC alone. Abbreviations: 2LCTX – 2^nd^-line chemotherapy, BSC – best supportive care.

After progression or discontinuation of 1^st^-line chemotherapy, 42 patients (53%) were treated with best-supportive-care (BSC), and 38 patients (48%) were treated with BSC and 2^nd^-line chemotherapy (2LCTX). In terms of 2LCTX, most patients received fluoropyrimidine monotherapy (n = 26 (68%)). Eight (21%) and 4 (11%) patients were treated with fluoropyrimidine-based combination chemotherapy or other regimens, respectively (Table 1). Median OS was 12.1 months in the 2LCTX + BSC group, and 2.7 months in the BSC group, respectively. The 3-, 6-, 12- and 18-months OS estimates were 92%, 77%, 53%, and 23% in the 2LCTX + BSC group, and 44%, 29%, 21%, and 14% in the BSC group (log-rank p = 0.0003, Fig. 1). In univariable Cox regression, 2LCTX + BSC was associated with a 0.4-fold lower relative risk of death-from-any-cause than BSC alone (Hazard ratio (HR) = 0.36, 95%CI: 0.20–0.64, p = 0.001).

### Derivation of the IPTW

Importantly, patients in the 2LCTX + BSC had a significantly higher prevalence of favorable prognostic factors (Table 1). For example, the median Karnofsky Index was 90% in 2LCTX + BSC, and 80% in BSC (rank-sum p = 0.0001; standardized mean difference (SMD) = 1.21, with SMDs >0.20 indicating a potentially important imbalance between study groups). Further, patients in the 2LCTX + BSC group had, among others, lower CRP levels (SMD = 0.45), lower alkalic phosphatase levels (SMD = 0.34) and lower bilirubin levels (SMD = 0.49) than patients in the BSC group, and all these variables were associated with a more favorable overall survival experience (Supplementary Table 1). Because this is a major source of bias for the 2LCTX + BSC vs. BSC comparison, we constructed a propensity score (PS) to predict probabilities of treatment assignment conditional on covariates at baseline. We constructed the PS using a multivariable logistic regression model, in which we included a broad set of variables irrespective of their association with OS (Supplementary Table 2). The distribution of the PS (Supplementary Fig. 2A) covered the whole probability range from 0 to 1, and was then transformed into the IPTW according to the inverse of the probability of receiving the treatment that the patient actually received (Supplementary Fig. 2B). Re-weighing of the data with the IPTW removed most imbalances of baseline covariates between the two treatment groups (Table 1). For example, IPTW-weighing reduced the SMDs for the key prognostic variables (1) Karnofsky Index from 1.21 to 0.31, (2) alkalic phosphatase from 0.34 to 0.01, and (3) bilirubin from 0.49 to 0.19, respectively. Although IPTW-weighing did not fully reduce imbalances below the pre-specified SMD threshold of 0.20 for a small number of variables such as haemoglobin, we considered these balance diagnostics to be indicative of an adequate propensity score model.

### IPTW-weighted analysis of overall survival according to treatment group

**Figure 2:**
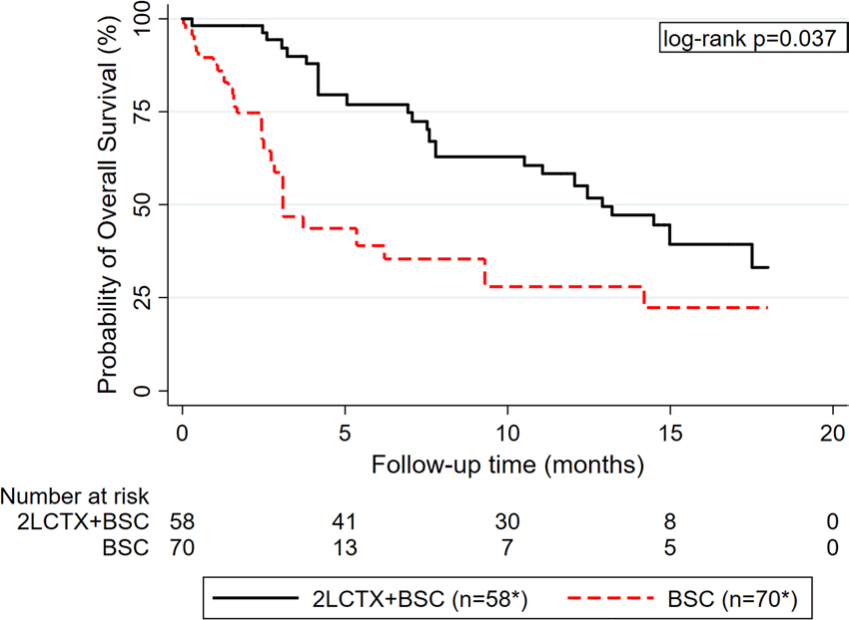
IPTW-weighted Kaplan-Meier curves of overall survival according to treatment assignment to 2LCTX + BSC versus BSC alone. *Number of patients represent the number in the synthetic pseudo-population generated by the IPTW. Abbreviations: IPTW – Inverse probability of treatment weight, 2LCTX – 2^nd^-line chemotherapy, BSC – best supportive care.

After IPTW weighting of the data, median OS was 12.9 months in the 2LCTX + BSC group, and 3.1 months in the BSC group, respectively. The 3-, 6-, 12- and 18-month OS estimates were 94%, 77%, 58%, and 33% in the 2LCTX + BSC group, and 59%, 39%, 28%, and 22% in the BSC group (log-rank p = 0.037, Fig. 2). In IPTW-weighted Cox regression, 2LCTX + BSC was associated with a 0.4-fold lower relative risk of death-from-any-cause than BSC alone (Hazard ratio (HR) = 0.40, 95%CI: 0.17–0.95, p = 0.037). To further increase the efficiency of this estimate, we applied a backward selection algorithm with a p = 0.10 threshold for removal to construct a multivariable model. This process selected 2LCTX, C-reactive protein, and bilirubin for multivariable analysis, and also here, 2LCTX remained associated with a better OS experience (Adjusted HR for 2LCTX = 0.38, 95%CI: 0.17–0.84, p = 0.017; Adjusted HR for CRP per 10 mg/dL increase = 1.17, 95%CI: 1.13–1.21, p < 0.0001; Adjusted HR for bilirubin per 1 mg/dL increase = 1.12, 95%CI: 1.03–1.22, p = 0.007). In a sensitivity analysis using the “trimmed” IPTW, a comparable IPTW-adjusted relative risk estimate was observed (IPTW-adjusted HR for 2LCTX-BSC vs. BSC = 0.48 (95%CI: 0.23–0.99, p = 0.048)).

### Exploring potential time-dependencies of 2LCTX benefit

**Figure 3:**
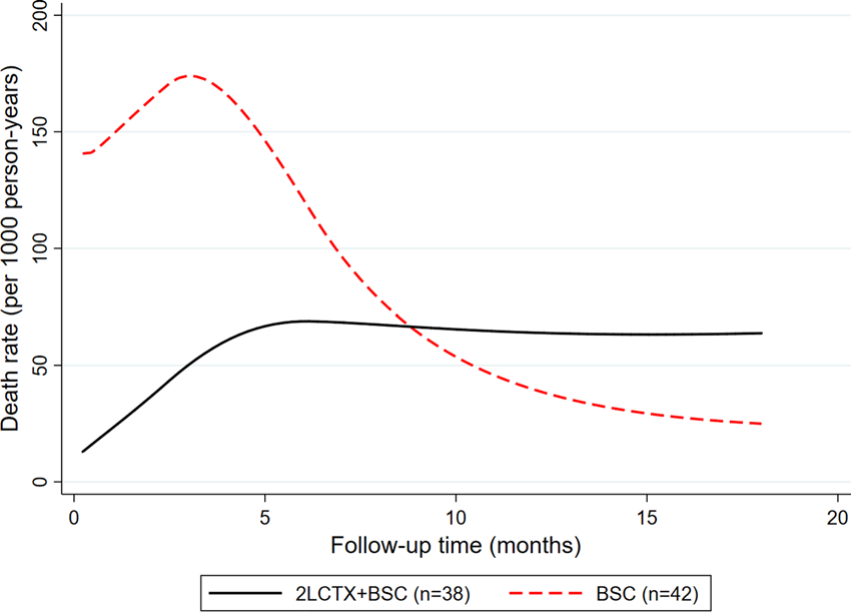
Hazards of death-from-any-cause according to treatment assignment to 2LCTX + BSC vs. BSC alone. Hazard curves were predicted from a flexible parametric survival model (log(cumulative hazard) scale) with 3 degrees of freedom for the time-invariant treatment variable and 2 degrees of freedom for the time-varying treatment variable. Abbreviations: 2LCTX – 2^nd^-line chemotherapy, BSC – best supportive care.

**Figure 4:**
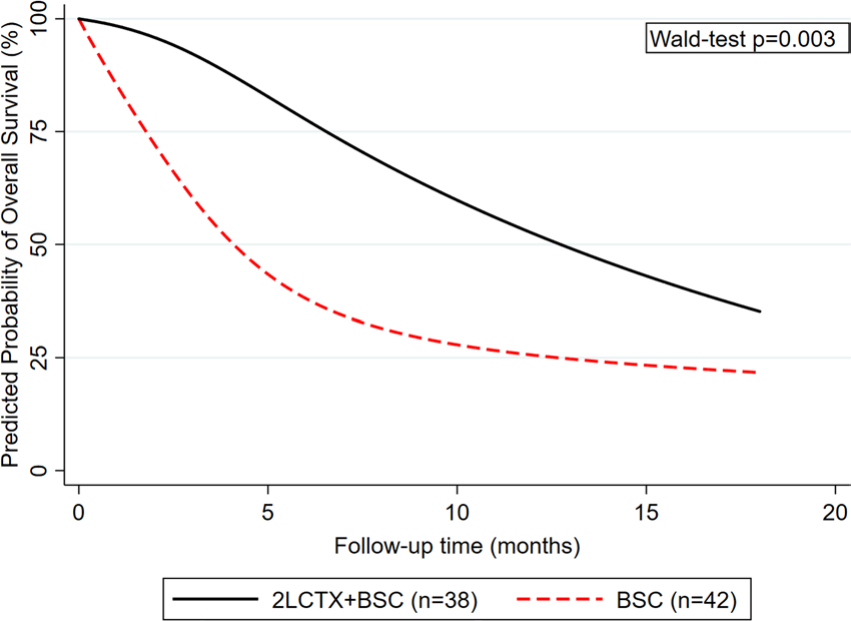
Predicted probability of overall survival according to treatment assignment to 2LCTX + BSC vs. BSC alone. Survival curves were predicted from a flexible parametric survival model (log(cumulative hazard) scale) with 3 degrees of freedom for the time-invariant treatment variable and 2 degrees of freedom for the time-varying treatment variable. Abbreviations: 2LCTX – 2^nd^-line chemotherapy, BSC – best supportive care.

We observed strong evidence for a violation of the proportional hazards association (Schoenfeld test p ≤ 0.005 for both unadjusted and IPTW analyses, respectively). Indeed, non-proportional analysis of mortality hazards using flexible parametric modeling showed the rate of death was much higher during the first few months of follow-up in the BSC only group. However, the rate of death increased over time in the 2LCTX + BSC group, which ultimately lead to a crossing of the two death rates at around 8 months of follow-up (Fig. 3). This non-proportionality was confirmed in IPTW-weighted Cox regression, where we observed a time-dependent association between 2LCTX and OS benefit (Hazard ratio for interaction between 2LCTX and linear follow-up in months = 1.25, 95%CI: 1.01–1.55, p = 0.036). Consistent with a weakening “effect” of 2LCTX over time, the IPTW-weighted HRs for 2LCTX + BSC vs. BSC alone were 0.23 (p = 0.002), 0.32 (p = 0.007) and 0.40 (p = 0.037) for prediction horizons of 6 months, 12 months, and 18 months of follow-up, respectively. Finally, to explicitly allow for these time-dependencies, we fitted a fully parametric, IPTW-weighted survival model with restricted cubic splines on the log(cumulative hazard) scale (3 degrees of freedom for the time-invariant and 2 degrees of freedom for the time-dependent “effect” of 2LCTX on OS). In this model, the association between 2LCTX and a lower relative risk of death prevailed (HR = 0.22, 95%CI: 0.08–0.60, p = 0.003, Fig. 4).

### Exploring potential predictive markers for 2LCTX benefit

Potential effect modifications between 2LCTX benefit and selected baseline covariables were examined by fitting an interaction between treatment assignment and the covariate of interest within IPTW-weighted Cox models (Table 2). In this analysis, “effect” estimates of 2LCTX were highly similar between patients who (1) did and did not respond to 1^st^ line chemotherapy, and (2) had bilirubin levels ≤ and >the 50^th^ percentile of its distribution. The benefit of 2LCTX + BSC appeared to be higher in patients with gallbladder carcinoma than in other aBTC subentities. Patients with elevated CRP, ECOG performance status of 1–2, and synchronous aBTC had numerically but not statistically significantly more favorable hazard ratios for 2LCTX benefit.

**Table 2:** Subgroup analyses of 2LCTX benefit in aBTC Table 2.

	Hazard ratio	95%CI	Interaction p-value
ECOG: 0	0.89	0.24–3.39	0.237
ECOG: 1–2	0.37	0.13–1.08	
Synchronous aBTC	0.30	0.07–1.20	0.524
Metachronous aBTC	0.45	0.16–1.24	
No response during 1stline CTX	0.41	0.16–1.04	0.159
Response during 1stline CTX	0.24	0.03–1.73	
Bilirubin ≤ 50^th^ percentile	0.40	0.13–1.25	0.813
Bilirubin > 50^th^ percentile	0.58	0.24–1.36	
C-reactive protein ≤ 50^th^ percentile	0.54	0.15–1.88	0.349
C-reactive protein > 50^th^ percentile	0.32	0.15–0.70	
aBTC: Gallbladder	0.05	0.01–0.39	0.020
aBTC: Intrahepatic CCC	0.64	0.22–1.88	
aBTC: Others	1.08	0.28–4.24	

Subgroup analyses of 2LCTX benefit in aBTC. Hazard ratios and interaction p-values were derived from IPTW-weighted Cox regression models of overall survival. Abbreviations: 2LCTX – 2^nd^-line chemotherapy, aBTC – Advanced biliary tract cancer, 95%CI – 95% confidence interval, ECOG – Eastern Cooperative Oncology Group performance status, CTX - chemotherapy

### Sensitivity analysis - Time-to-progression in the 2LCTX+BSC group and further line therapies

**Figure 5:**
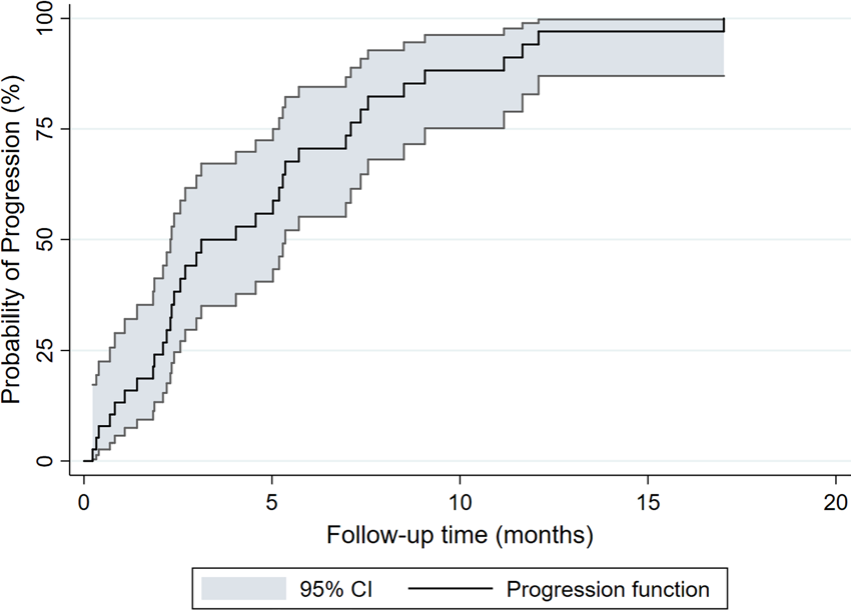
Time-to-progression (TTP) in the 2LCTX + BSC group (n = 38). TTP with 95% confidence bands (gray shadowing) was estimated with an inverse Kaplan-Meier estimator.

Thirty-five (92%) of the 38 patients in the 2LCTX + BSC group had documented disease-progression at the data cut-off. The median time-to-progression (TTP) was estimated at 4.0 months (95%CI: 2.3–5.4, Fig. 5). Seventy-five percent of the cohort remained free-from-progression for at least 2.1 months (95%CI: 0.6–2.6), and 25% of the cohort remained free-from-progression for at least 7.1 months (95%CI: 5.2–9.1).

After progression on 2^nd^-line therapy, 11 patients in the 2LCTX + BSC group also received a 3^rd^ treatment line (n = 1 Gemcitabine, n = 1 capecitabine, n = 4 FOLFOX, n = 2 erlotinib, n = 1 temsirolimus within a genomically-guided therapy trial, n = 1 docetaxel, and n = 1 trastuzumab). One of these 11 patients even received a 4^th^ line of treatment (with FOLFIRI).

### Sensitivity analysis – A simplified propensity score

Using backward elimination, we reduced the propensity score model to 8 predictor variables (Supplementary Table 4), but the subsequent simplified IPTW did not as good balance as the original 20-variable propensity score (Table 1). The association between 2LCTX + BSC and favorable OS prevailed after weighting with the simplified IPTW (Hazard ratio = 0.30, 0.12–0.72, p = 0.007). Median IPTW-weighted OS was 15.0 months in the 2LCTX + BSC group, and 3.1 months in the BSC group, respectively (log-rank p = 0.007, Supplementary Fig. 3).

### Sensitivity analysis – Landmark analysis

The median time to initiation of 2LCTX was 8 days [25^th^–75^th^ percentile: 1–32], which has the potential for introducing so-called immortal time bias into our comparison of 2LCTX + BSC vs. BSC. Thus, a landmark analysis was performed with a landmark date at 28 days, representing a reasonable “clinical window” for commencing 2LCTX, and 28 patients received 2LCTX within this time frame. Six-, 12-, and 18-month Kaplan-Meier OS estimates were 78%, 38%, and 10% in the 28 patients who received 2LCTX within the first 28 days, and 36%, 30%, and 20% in the remaining 52 patients who did not receive 2LCTX within this time-frame, respectively (Mantel-Byar p = 0.032, see figure below). Finally, we treated initiation of 2LCTX as a time-dependent variable, thus also controlling for potential immortal time bias. In this analysis, 2LCTX + BSC treated as a time-dependent variable within an unweighted Cox model was associated with a 0.5-fold reduction in the risk of death (Hazard Ratio (HR) = 0.53, 95%CI: 0.29–0.95, p = 0.034).

## Discussion

Randomized data on the benefit of 2LCTX in addition to BSC alone in patients with aBTC are lacking. In this study, we performed a propensity-score weighted analysis of 18-month observational data from 80 aBTC patients to obtain estimates on the association between 2LCTX and OS. We found that patients receiving 2LCTX + BSC had a significantly better OS experience than patients with BSC alone. However, this “univariable” finding was highly confounded by the non-random selection of patients with favorable covariables into the 2LCTX + BSC group. To account for this bias, we re-weighted the data according to probabilities of assignment to 2LCTX conditional on covariates. Upon this adjustment, the favorable association between 2LCTX and 18-month OS became slightly weaker but prevailed. Additional multivariable adjustment for key prognostic variables such as performance status and serum bilirubin levels did not materially alter this estimate. Importantly, we found evidence for a time-dependency of 2LCTX, indicating that the benefit of this intervention slightly weakened over time. Nonetheless, the overall benefit of 2LCTX prevailed also after fully taking into account this time-dependency using flexible parametric models. In subgroup analyses, the potential benefit of 2LCTX appeared to be consistent across several subgroups defined by clinical and laboratory variables such as performance status, treatment response during 1^st^-line chemotherapy, and bilirubin levels. Within the limitations of an observational study, these data support the concept that 2LCTX + BSC delays death in patients with aBTC.

A fundamental question for oncologists treating patients with aBTC after failure of first-line chemotherapy is whether 2LCTX improves survival over BSC alone. To our knowledge, this question has not been systematically addressed before. In the absence of these data, several retrospective single-arm studies have reported favorable OS outcomes in aBTC patients treated with systemic chemotherapy in the second-line setting. For example, Kim and colleagues recently observed a median OS of 6.5 months in 321 patients treated with fluoropyrimidine-based mono- or polychemotherapy^15^. Other median OS estimates in patients undergoing 2LCTX for aBTC ranged from approximately 7 months in the large cooperative studies by Brieau *et al*. (6.5 months), Fornaro *et al*. (6.6 months) and Walter *et al*. (7.5 months)^10,11,17^, and the meta-analysis by Lamarca *et al*. (7.2 months)^23^, to 13.8 months in a single-center chart review study by Rogers and colleagues^13^. The median OS of 12 months observed in this study for patients who received 2LCTX compares well with these previous reports. Analysis of time-to-progression in the 2LCTX + BSC group revealed a relatively favorable median TTP interval of 4 months, with 25% of patients remaining free-from-progression for at least 7 months. Importantly, this median TTP interval was longer than the median OS interval of 2.7 months in the BSC only group. Together, these data provide preliminary support for the clinically-plausible concept that aBTC patients may benefit from 2LCTX in addition to BSC. Moreover, theoretical support for this concept comes from Hagen-Pouiseuille’s law, which states that only a small decrease in biliary lumen due to local tumor progression may dramatically reduce biliary flow^7^. The resulting biliary stenosis is an established risk factor for life-threatening infection, and often leads to discontinuation of chemotherapy and morbidity subsequent to repeated interventional procedures for restoring biliary patency. Thus, 2LCTX may not only delay the adverse effects of metastatic tumor spread and progression at distant organs, but may also prolong the time-to-biliary-stenosis and its adverse effects on morbidity and survival^24^.

However, previous retrospective single-arm analyses of highly-selected patients can obviously not answer the underlying systematic question on whether 2LCTX has any benefit at all over BSC alone in a general population of aBTC patients. This is also highlighted by a recent systematic review, which concludes that there is currently insufficient evidence to recommend 2LCTX in aBTC^23^. In this study, we aimed to address this important clinical question using an inverse-probability-of-treatment-weighted comparative effectiveness analysis of observational data. This approach was necessary to account for the large amount of selection bias likely affecting such analyses^25^. Given the statistical assumptions underlying this propensity-score-based approach are met, such an analysis generates a synthetic pseudo-population whose treatment assignment is independent of covariates, hence mimicking randomization^22^. In our study, “naïve” analysis of OS outcomes was consistent with a potentially large benefit of 2LCTX + BSC over BSC alone, both from an absolute (OS estimates) and relative (hazard ratios) perspective. IPTW weighing was performed according to best practice recommendations^26^, and removed most covariate imbalances between these two study groups. The beneficial association between 2LCTX and favorable OS prevailed upon IPTW weighting of time-to-death-data both with respect to magnitude and strength of association. Interestingly, we found that the beneficial “effect” of 2LCTX slightly weakened over time. Indeed, the survival curves of the two treatment groups approached over time. We took this time-dependency into account by specifically modeling non-proportionality of hazards within flexible parametric models^27^, and the beneficial association of 2LCTX with OS also prevailed in this analysis. Synoptically, this suggests that 2LCTX in this setting is an archetypical *palliative* treatment which delays death but not necessarily leads to a higher proportion of long-term survivors. Physicians should take this into account when discussing second-line options with their patients.

An important aspect of clinical cancer research is to identify predictive markers for treatment response^28^. In the second-line aBTC setting, such markers may inform clinical decision making by stratifying patients according to potentially high or low likelihoods of benefiting from 2LCTX and thus facilitate the decision in favor or against 2LCTX^29^. We have performed such an analysis by fitting interactions between selected baseline variables and OS benefit from 2LCTX. Importantly, patients who did and did not respond to 1^st^-line chemotherapy appeared to have a similar benefit from 2LCTX. Moreover, this also applied to patients with bilirubin levels below and above the 50^th^ percentile of this covariate’s distribution. This allows us to carefully speculate that neither lack of objective response during 1^st^ line chemotherapy nor moderate biliary stenosis as indicated by elevated bilirubin levels should preclude oncologists from considering 2LCTX. Although not reaching statistical significance, we observed numerically higher relative risk reductions of mortality with 2LCTX in patients with elevated C-reactive protein. This generates the hypothesis that patients with more “inflamed” biliary tract cancers may particularly benefit from 2LCTX. This findings should be considered in the context of poor general prognosis for patients with unfavorable alterations in markers of systemic inflammatory response^30,31^. Finally, patients with gallbladder carcinoma appeared to have a greater benefit from 2LCTX + BSC than patients with other subentities of aBTC. However, given the small sample size and “underpowering” of these hypothesis-generating subgroup analyses, we urge readers to interpret these subgroup findings with the necessary caution until validated in other cohorts.

Finally, we want to mention several limitations of this study. First, due to the heterogeneity of 2LCTX regimens in our population and the relatively low number of patients treated with some of these individual 2LCTX regimens, we cannot provide robust estimates on the most optimal chemotherapy regimen in this setting. Second, the potentially large magnitude of “effect” of 2LCTX in our study may not exclusively be attributable to a “true” benefit from 2LCTX, but may also be due to from residual confounding not removed by the IPTW. Importantly, the validity of an IPTW analysis depends on the difficult-to-test assumption that the propensity score model is correctly specified and does not omit unmeasured confounders^26^. We have addressed this issue by balance diagnostics after IPTW weighting and careful multivariable adjustment. Moreover, we estimated TTP data, which were relatively favorable and further support the concept that the OS experience of patients in the 2LCTX + BSC group is at least partly attributable to a “chemo effect.” Nonetheless, it remains a limitation of our study that inverse-probability-of-treatment-weighting did not fully reduce all differences in baseline covariates between the two study groups. For example, important prognostic variables such as haemoglobin and albumin still showed SMDs well above the usual threshold of 0.2 after IPTW, and we thus cannot rule out some residual confounding which might have biased our results in favor of 2LCTX. Another potential residual confounder beyond IPTW are further-line treatments. Indeed, 11 patients in the 2LCTX + BSC group went on to receive 3^rd^-line treatments after progression on 2LCTX, and one patient even received a 4^th^ treatment line. Thus, the relatively favorable OS experience of the 2LCTX + BSC group may also be in part mediated by potential activity of further-line treatments. Third, we included a diverse spectrum of BTC histologies from intrahepatic cholangicarcinoma to cancer-of-unkown-primary (CUP-CCC) with cholangiocellular differentiation. Although these subentities are all classified as biliary tract cancers, translational studies have shown that they can substantially differ with respect to molecular features^32^. These differences may have an impact on 2LCTX benefit, and we found that patients with gallbladder cancer may have a particularly high benefit from 2LCTX. But also here, low numbers of patients within some histologic subentities precluded definitive conclusions on 2LCTX between these subentities. Fourth, our dataset does not yet include data on interventional procedures such as biliary stenting, which may modify the survival experience of this patient population^24^. Fifth, in the absence of validated cut-offs, we empirically dichotomized our patients at the 50^th^ percentile of continuous variables for predictive biomarker analysis. Higher or lower cut-offs may have yielded different subgroup estimates of 2LCTX benefit, but we refrained from examining other cut-offs in order not to further inflate the type I error rate. Sixth, quality-of-life is a paramount issue for patients suffering from a lifetime-limiting disease such as aBTC after first-line chemotherapy^6^. However, due to the retrospective nature of this study, data on quality-of-life were not available to us. Finally, our treatment comparison may be subject to so-called immortal time bias, as patients who may have received 2LCTX may have died in the interval from disease progression to start of 2LCTX. However, we have performed sensitivity analyses with landmark-analysis and models treating 2LCTX as a time-dependent variable, thus strongly reducing the potential for unaccounted immortal time bias.

## Conclusion

Within the limitations of an observational cohort study, these data support the concept that 2LCTX + BSC is associated with an overall survival benefit over BSC alone in patients with aBTC after failure of first-line chemotherapy. This benefit slightly weakens over time, but appears to be consistent across several subgroups defined by clinical and laboratory variables such as performance status, treatment response during 1^st^-line chemotherapy, and moderate biliary stenosis as indicated by elevated serum bilirubin levels. Until randomized evidence becomes available in the future, our findings provide guidance to oncologists and their aBTC patients for treatment decision making in the second-line setting. Future studies should address the benefit of 2LCTX in aBTC within a randomized setting, and identify those patient subgroups with the highest benefit from 2LCTX.

## Methods

### Study Population and Design

In this single-center, observational, historical cohort study, we included all consecutive patients with histologically-confirmed aBTC who completed 1LCTX at the Division of Oncology, Medical University of Graz, Austria (n = 80). This patient population was drawn from the greater population of patients with non-advanced and advanced BTC treated at our Department between 2003 and 2016 (n = 185). From this population, we excluded patients who did not progress after resection/treatment in curative intent (n = 79), patients who did not receive 1^st^-line chemotherapy (n = 18), and patients who were lost-to-follow-up (n = 1). Further 7 patients were excluded because they died during 1^st^-line chemotherapy (n = 3), or due to missing data already in 1^st^-line chemotherapy (n = 4). Baseline and outcome data for the 80 remaining patients were retrieved retrospectively from a prospectively-maintained in-house electronic health care database as reported previously^33,34^. The baseline date was defined as the date of progression in 1^st^-line chemotherapy for both groups. In case patients did not progress during 1^st^-line chemotherapy, but 1^st^-line chemotherapy was terminated and BSC alone initiated due to poor performance status, we selected the end date of the last chemotherapy cycle as the baseline date. Primary endpoint of this study was death-from-any-cause within 18 months of follow-up. Data collection and analysis was approved by the local institutional review board (Ethics Committee of the Medical University of Graz, Austria; document number No. 25–458 ex 12/13). All methods were performed in accordance with the relevant local and national guidelines and regulations.

### Statistical methods

All statistical analyses were performed using Stata (Windows version 14.0, Stata Corp., Houston, TX, USA). Differences in means and proportions between patients in the 2LCTX + BSC and BSC group were quantified using standardized mean differences (SMDs)^26^, and further evaluated with Wilcoxon’s rank-sum tests, χ^2^-tests, and Fisher’s exact tests, respectively. SMDs >0.20 were considered to indicate potentially relevant imbalance between the two study groups^26^. Median follow-up was computed with the reverse Kaplan-Meier estimator according to Schemper & Smith^35^, whereas OS was computed with the traditional Kaplan-Meier estimator. Log-rank tests were used for comparing overall survivor functions between the two study groups. For uni- and multivariable modeling of time-to-death-from-any-cause, Cox proportional hazards models were fitted. For each patient, the propensity score *e* was defined as the probability of receiving 2LCTX + BSC conditional on baseline covariates, and the inverse-probability-of-treatment-weight (IPTW) was defined as the inverse of the probability of receiving the treatment that the patient received^26^. We calculated the propensity score using a multivariable logistic regression model including all covariates reported in Supplementary Table 2. For this model, we imputed missing baseline covariates using a chained equations algorithm with 25 imputation datasets (Stata routine mi impute chained; a list with the conditional imputation models is available on request from the corresponding author, and a list with the variables used for multiple imputation is reported in Supplementary Table 3)^36^. To explore whether IPTW yielded balance on baseline variables between the two study groups, SMDs were re-estimated after weighing of the data with the IPTWs following best-practice recommendations^26^. IPTW-weighted Kaplan-Meier estimators and Cox proportional hazards models were fitted for analyzing the “imbalance-adjusted” association between 2LCTX and OS, respectively^22^. In a sensitivity analysis, we used a “trimmed” IPTW excluding patients <5^th^ and >95^th^ percentile of the IPTW’s distribution^37^. Because we observed strong evidence for a violation of the proportional hazards assumption in the time-to-death analysis (as indicated by Schoenfeld test p-values and the interaction hazard ratio between study group and follow-up time), analyses were re-performed with flexible parametric models allowing for time-varying associations of study group and time-to-death (Stata routine stpm2)^27^. Subgroup analyses were performed by fitting interactions between treatment assignment and the subgroup/covariate of interest. Here, interaction p-values (rather than subgroup p-values) were considered as measures for testing the hypothesis of a differential association of 2LCTX and OS between the pertinent subgroups^38^. In sensitivity analyses, we explored time-to-progression (TTP), a simplified IPTW based on a reduced propensity score model, and performed land-mark analyses to account for potential immortal-time bias. Time-to-progression (TTP), defined as the interval from the start of 2LCTX to the time of radiographic and/or clinical progression, was estimated in the 2LCTX + BSC group only using an inverse Kaplan-Meier estimator. For developing a simplified IPTW, we used the original 20-covariate PS model and then performed a backward elimination to 8 variables in total (excluding one-by-one the variables with the smallest strength of association as indicated by the t-statistic in the logistic regression) in order to obtain a PS model with 10 patients per predictor variable. This 8-variable model is reported in Supplementary Table 4. In landmark analysis, we computed overall survival from a landmark date of 28 days (i.e. 4 weeks after progression) for patients who did and did not receive 2LCTX within this time frame. Estimates were compared using the Mantel-Byar method. Moreover, in a final sensitivity analysis, we treated initiation of 2LCTX as a time-dependent variable, thus also incorporating potential immortal time bias. The full analysis code is available on request from the corresponding author.

### Ethics approval and consent to participate

This study was approved by the local institutional review board (Ethics Committee of the Medical University of Graz, Austria; document number No. 25–458 ex 12/13).

## Supplementary information

Supplementary Data

## Data Availability

The dataset generated and analyzed during the current study is not publicly available because public data sharing is not covered by the approval of the local ethics committee. However, data may be shared under a data sharing agreement upon reasonable request to the corresponding author.
