# Supplementary material for: Benefit of second-line systemic chemotherapy for advanced biliary tract cancer: A propensity score analysis
Source: Sci Rep. 2019 Apr 3;9:5548. doi: 10.1038/s41598-019-42069-1 (PMC6447553; doi:10.1038/s41598-019-42069-1)
Supplement: Supplementary file 1 — Supplementary Data [file 41598_2019_42069_MOESM1_ESM.docx]

**SUPPLEMENTARY DATA FILE**

**Benefit of second-line systemic chemotherapy for advanced biliary tract cancer: A propensity score analysis**

Florian Moik, MD,^1,2^ Jakob M. Riedl, MD,^1^ Thomas Winder, MD,^3^ Angelika Terbuch, MD,^1^ Christopher H. Rossmann, MD,^1^ Joanna Szkandera, MD,^1^ Prof. Thomas Bauernhofer, MD,^1^ Anne-Katrin Kasparek, MD,^1^ Renate Schaberl-Moser, MD,^1^ Andreas Reicher, MSc,^1,4^ Felix Prinz, MSc,^1^ Prof. Martin Pichler, MD MSc,^1,5^ Prof. Herbert Stöger, MD,^1^ Michael Stotz, MD,^1^ Prof. Armin Gerger, MD MBA,^1,6^ and Florian Posch, MD MSc^1,6*^

^1^ Division of Oncology, Department of Internal Medicine, Medical University of Graz, Auenbruggerplatz 15, 8036 Graz, Austria

Florian Moik: [florian.moik@stud.medunigraz.at](mailto:florian.moik@stud.medunigraz.at) Jakob Riedl: [j.riedl@stud.medunigraz.at](mailto:j.riedl@stud.medunigraz.at) Angelika Bezan: [angelika.terbuch@medunigraz.at](mailto:angelika.terbuch@medunigraz.at) Christopher H. Rossmann: [ChristopherHerbert.Rossmann@klinikum-graz.at](mailto:ChristopherHerbert.Rossmann@klinikum-graz.at) Joanna Szkandera: [joanna.szkandera@medunigraz.at](mailto:joanna.szkandera@medunigraz.at) Thomas Bauernhofer: [thomas.bauernhofer@medunigraz.at](mailto:thomas.bauernhofer@medunigraz.at) Anne-Katrin Kasparek: [Anne-Katrin.Kasparek@klinikum-graz.at](mailto:Anne-Katrin.Kasparek@klinikum-graz.at) Renate Schaberl-Moser: [renate.schaberl-moser@medunigraz.at](mailto:renate.schaberl-moser@medunigraz.at) Felix Prinz: [felix.prinz@medunigraz.at](mailto:felix.prinz@medunigraz.at) Martin Pichler: [martin.pichler@medunigraz.at](mailto:martin.pichler@medunigraz.at) Herbert Stöger: [herbert.stoeger@medunigraz.at](mailto:herbert.stoeger@medunigraz.at) Michael Stotz: [michael.stotz@medunigraz.at](mailto:michael.stotz@medunigraz.at) Armin Gerger: [armin.gerger@medunigraz.at](mailto:armin.gerger@medunigraz.at) Florian Posch: [florian.posch@medunigraz.at](mailto:florian.posch@medunigraz.at)

^2^ Clinical Division of Haematology & Haemostaseology, Department of Medicine I, Währinger Gürtel 18-20, 1090 Vienna, Austria

Florian Moik: [florian.moik@meduniwien.ac.at](mailto:florian.moik@meduniwien.ac.at)

^3^ Division of Oncology, Department of Internal Medicine II, Academic Teaching Hospital Feldkirch, Carinagasse 47, 6800 Feldkirch, Austria

Thomas Winder: [Thomas.Winder@gmx.at](mailto:Thomas.Winder@gmx.at)

^4^ Research Center for Molecular Medicine (CeMM) of the Austrian Academy of Sciences, Lazarettgasse 14, 1090 Vienna, Austria

Andreas Reicher: [AReicher@cemm.oeaw.ac.at](mailto:AReicher@cemm.oeaw.ac.at)

^5^ Department of Experimental Therapeutics, The University of Texas MD Anderson Cancer Center, 1901 East Road, Room 3SCR4.3424, Houston, Texas 77054, USA

Martin Pichler: [mpichler@mdanderson.org](mailto:mpichler@mdanderson.org)

^6^ Center for Biomarker Research in Medicine (CBmed), Stiftingtalstrasse 5, 8010 Graz, Austria

Armin Gerger: [armin.gerger@cbmed.at](mailto:armin.gerger@cbmed.at)

Florian Posch: [florian.posch@cbmed.at](mailto:florian.posch@cbmed.at)

**Supplementary Tables**

**Supplementary Table 1**

| **Supplementary Table 1. Univariable predictors of time-to-death in the overall study population.** | | | |
| --- | --- | --- | --- |
|  | | | |
| **Variable** | **Hazard Ratio** | **95%CI** | **p** |
|  |  |  |  |
| **Demographics** |  |  |  |
| Age (per 5 years increase) | 0.93 | 0.80-1.07 | 0.314 |
| Female Gender | 1.24 | 0.70-2.18 | 0.463 |
| BMI (per 5 kg/m² increase) | 0.76 | 0.54-1.06 | 0.100 |
| History of smoking | 1.15 | 0.61-2.15 | 0.669 |
| Charleson Comorbidity Index  (per 1 point increase) | 1.04 | 0.91-1.19 | 0.534 |
| Synchronous aBTC | 1.95 | 1.08-3.53 | 0.028 |
| ECOG 1-2 | 2.59 | 1.33-5.02 | 0.005 |
| **Tumor location** | / | / | / |
| ---Gallbladder | Ref. | Ref. | Ref. |
| ---Intrahepatic | 0.89 | 0.45-1.75 | 0.741 |
| ---Perihilar/Klatskin | 0.69 | 0.28-1.73 | 0.435 |
| ---Distal/Ampulla | 0.27 | 0.10-0.72 | 0.008 |
| ---CUP-CCC* | NE | NE | NE |
| **Tumor grade** | / | / | / |
| ---G3 | 1.62 | 0.90-2.90 | 0.107 |
| **1^st^ line CTX data** | / | / | / |
| ---Cisplatin/Gemcitabine | Ref. | Ref. | Ref. |
| ---Gemcitabine mono | 0.56 | 0.29-1.05 | 0.072 |
| ---Other CTX regimens | 0.44 | 0.19-1.02 | 0.056 |
| **Laboratory parameters** | / | / | / |
| Haemoglobin (per 1g/dL increase) | 0.67 | 0.55-0.82 | <0.0001 |
| Leukocyte count (per 1G/L increase) | 1.16 | 1.09-1.23 | <0.0001 |
| Neutrophil count (per 1G/L increase) | 1.06 | 1.03-1.09 | <0.0001 |
| Lymphocyte count (per 1G/L increase) | 0.93 | 0.90-0.97 | <0.0001 |
| Platelet count (per 50G/L increase) | 0.95 | 0.87-1.04 | 0.260 |
| Neutrophil-Lymphocyte ratio  (NLR, per 1 unit increase) | 1.17 | 1.11-1.24 | <0.0001 |
| C-reactive protein (per doubling) | 1.64 | 1.35-1.99 | <0.0001 |
| Bilirubin (per doubling) | 1.47 | 1.24-1.75 | <0.0001 |
| Gamma-GT (per doubling) | 1.29 | 1.08-1.53 | 0.004 |
| Alkalic Phosphatase (per doubling) | 1.92 | 1.46-2.52 | <0.0001 |
| Creatinine (per 1mg/dL increase) | 0.49 | 0.16-1.49 | 0.211 |
| AST (per doubling) | 1.57 | 1.17-2.11 | 0.003 |
| ALT (per doubling) | 1.36 | 1.05-1.75 | 0.020 |
| Albumin (per 1g/dL increase) | 0.38 | 0.21-0.67 | 0.001 |
| CEA (per doubling) | 1.15 | 0.99-1.34 | 0.072 |
| CA199 (per doubling) | 1.21 | 1.08-1.36 | 0.001 |

**Supplementary Table 2**

| **Supplementary Table 2. Propensity score model for treatment assignment to 2LCTX+BSC versus BSC alone.** | | | |
| --- | --- | --- | --- |
|  | | | |
| **Variable** | **Odds Ratio** | **95%CI** | **p** |
|  |  |  |  |
| **Demographics** |  |  |  |
| Age (per 1 year increase) | 0.88 | 0.70-1.10 | 0.255 |
| Male Gender | 3.55 | 0.26-48.47 | 0.342 |
| History of smoking | 0.16 | 0.01-2.27 | 0.174 |
| Charleson Comorbidity Index  (per 1 point increase) | 1.02 | 0.66-1.56 | 0.945 |
| Metachronous aBTC | 11.37 | 0.44-293.24 | 0.141 |
| ECOG 1-2 | 0.17 | 0.01-3.94 | 0.267 |
| **Tumor parameters** | / | / | / |
| ---Gallbladder | Ref. | Ref. | Ref. |
| ---Intrahepatic | 3.18 | 0.21-48.45 | 0.405 |
| ---Perihilar/Klatskin | 363.35 | 0.14-960191.60 | 0.141 |
| ---Others | 33.09 | 0.70-1557.75 | 0.075 |
| Tumor grade G3 | 0.35 | 0.02-7.22 | 0.493 |
| **1^st^ line CTX data** | / | / | / |
| ---Cisplatin/Gemcitabine | Ref. | Ref. | Ref. |
| ---Gemcitabine mono | 0.41 | 0.02-8.12 | 0.558 |
| ---Other CTX regimens | 0.38 | 0.01-40.01 | 0.686 |
| Objective response in 1^st^ line CTX | 0.01 | 0.00-0.82 | 0.043 |
| CTX cycles in 1^st^ line  (per 1 cycle increase) | 1.39 | 0.76-2.55 |  |
| **Laboratory parameters** | / | / | / |
| Haemoglobin (per 1g/dL increase) | 1.61 | 0.59-4.40 | 0.353 |
| Neutrophil count (per 1G/L increase) | 0.84 | 0.50-1.43 | 0.5252 |
| Platelet count (per 1G/L increase) | 1.01 | 1.00-1.01 | 0.106 |
| C-reactive protein  (per 1mg/dL increase) | 1.01 | 0.96-1.06 | 0.771 |
| Bilirubin (per 1mg/dL increase) | 0.78 | 0.39-1.57 | 0.493 |
| Gamma-GT (per 1unit/L increase) | 1.00 | 1.00-1.01 | 0.403 |
| Alkalic Phosphatase  (per 1unit/L increase) | 1.00 | 0.99-1.01 | 0.637 |
| Albumin (per 1g/dL increase) | 1.73 | 0.20-14.70 | 0.613 |
| CA199 (per 1 unit increase) | 1.00 | 1.00-1.00 | 0.343 |

**Supplementary Table 3**

| **Supplementary Table 3. Variables used for multiple imputation with n=25 imputation datasets.** | | | |
| --- | --- | --- | --- |
|  | | | |
| **Variable** | **N (complete)** | **N (imputed)** | **Imputation method** |
|  |  |  |  |
| **Demographics** |  |  |  |
| Age (years) | 80 | 0 | N/A |
| Male Gender | 80 | 0 | N/A |
| History of smoking | 72 | 8 | PMM |
| BMI (kg/m²) | 70 | 10 | LR |
| Charleson Comorbidity Index (points) | 80 | 0 | N/A |
| Metachronous aBTC | 71 | 9 | PMM |
| ECOG 1-2 | 60 | 20 | PMM |
|  |  |  |  |
| **Tumor parameters** |  |  |  |
| ---Gallbladder | 80 | 0 | N/A |
| ---Intrahepatic | 80 | 0 | N/A |
| ---Perihilar/Klatskin | 80 | 0 | N/A |
| ---Others | 80 | 0 | N/A |
| Non-adenocarcinoma histology | 80 | 0 | N/A |
| Tumor grade G3 |  |  |  |
|  |  |  |  |
| **1^st^ line CTX data** |  |  |  |
| ---Cisplatin/Gemcitabine | 80 | 0 | N/A |
| ---Gemcitabine mono | 80 | 0 | N/A |
| ---Other CTX regimens | 80 | 0 | N/A |
| Objective response in 1^st^ line CTX | 80 | 0 | N/A |
| CTX cycles in 1^st^ line | 79 | 1 | LR |
|  |  |  |  |
| **Laboratory parameters** |  |  |  |
| Haemoglobin (g/dL) | 78 | 2 | LR |
| Neutrophil count (G/L) | 78 | 2 | LR |
| Platelet count (G/L) | 78 | 2 | LR |
| C-reactive protein (mg/dL) | 77 | 3 | LR |
| Bilirubin (mg/dL) | 77 | 3 | LR |
| Gamma-GT (unit/L) | 77 | 3 | LR |
| Alkalic Phosphatase (unit/L) | 77 | 3 | LR |
| Albumin (g/dL) | 70 | 10 | LR |
| CA199 (units) | 43 | 32 | LR |

**Supplementary Table 4**

| **Supplementary Table 4. Simplified 8-variable propensity score model for treatment assignment to 2LCTX+BSC versus BSC alone.** | | | |
| --- | --- | --- | --- |
|  | | | |
| **Variable** | **Odds Ratio** | **95%CI** | **p** |
|  |  |  |  |
| **Demographics** |  |  |  |
| Age (per 1 year increase) | 0.93 | 0.85-1.01 | 0.102 |
| Metachronous aBTC | 6.23 | 1.12-34.59 | 0.037 |
| ECOG 1-2 | 0.13 | 0.02-0.74 | 0.002 |
|  |  |  |  |
| **Tumor parameters** | / | / | / |
| ---Gallbladder | Ref. | Ref. | Ref. |
| ---Intrahepatic | 4.74 | 0.78-28.94 | 0.092 |
| ---Perihilar/Klatskin | 144.78 | 4.12-5087.03 | 0.006 |
| ---Others | 33.69 | 3.05-372.44 | 0.004 |
|  |  |  |  |
| **1^st^ line CTX data** | / | / | / |
| Objective response in 1^st^ line CTX | 0.02 | 0.00-0.37 | 0.009 |
| CTX cycles in 1^st^ line  (per 1 cycle increase) | 1.32 | 0.93-1.87 | 0.124 |
|  |  |  |  |
| **Laboratory parameters** | / | / | / |
| Platelet count (per 1G/L increase) | 1.00 | 1.00-1.01 | 0.116 |
| CA199 (per 1 unit increase) | 1.00 | 1.00-1.00 | 0.093 |

# Supplementary Table legends

# Supplementary Table 1. Univariable predictors of time-to-death in the overall study population. Estimates were derived from univariable Cox regression models. Abbreviations: 95%CI – 95% confidence interval, p – Wald-test p-value, BMI – body mass index, ECOG – Eastern Cooperative Oncology Group performance status, CUP-CCC – cancer of unknown primary with cholangiocellular differentiation, CTX – chemotherapy, AST – Aspartate aminotransferase, ALT – Alanine aminotransferase, CEA – carcinoembryonic antigen, CA 19-9 – Cancer antigen 19-9.

# Supplementary Table 2. Propensity score model for treatment assignment to 2LCTX+BSC versus BSC alone. This model is a multivariable logistic regression model which uses data that were multiply imputed with a chained equations algorithm. Abbreviations: 2LCTX – 2^nd^-line chemotherapy, BSC – best supportive care, 95%CI – 95% confidence interval, p – Wald-test p-value, aBTC – Advanced biliary tract cancer, ECOG – Eastern Cooperative Oncology Group performance status, CTX – chemotherapy, AST – Aspartate aminotransferase, ALT – Alanine aminotransferase, CEA – carcinoembryonic antigen, CA 19-9 – Cancer antigen 19-9.

**Supplementary Table 3. Variables used for multiple imputation with n=25 imputation datasets.** Multiple imputation was performed with a chained equations algorithm. N(complete) reports the number of patients with an observed variable, and n(imputed) the number of patients for whom the missing value was imputed. Imputation methods were predictive mean matching and linear regression. Abbreviations: aBTC – Advanced biliary tract cancer, ECOG – Eastern Cooperative Oncology Group performance status, CTX – chemotherapy, CA19-9 – tumor marker Cancer Antigen 19-9, PMM – Predictive Mean Matching, LR – Linear Regression.

**Supplementary Table 4. Simplified 8-variable propensity score model for treatment assignment to 2LCTX+BSC versus BSC alone.** This model is a multivariable logistic regression model which uses data that were multiply imputed with a chained equations algorithm. The model was obtained by backward elimination of variables from the model reported in Supplementary Table 2 until 8 variables (10 patients per variable) were finally included. For multi-level categorical variables with n levels, we used a joint test on n-1 degrees of freedom to obtain a joint p-value for backward elimination. Abbreviations: 2LCTX – 2^nd^-line chemotherapy, BSC – best supportive care, 95%CI – 95% confidence interval, p – Wald-test p-value, aBTC – Advanced biliary tract cancer, ECOG – Eastern Cooperative Oncology Group performance status, CTX – chemotherapy, CA 19-9 – Cancer antigen 19-9.

# Supplementary figures

**Supplementary figure 1**

**Supplementary figure 2**

**Supplementary Figure 3**

**Supplementary Figure 4**

# Supplementary figure legends

**Supplementary Figure 1. 18-month overall survival experience of the study cohort (n=80).** The curve represents a crude Kaplan-Meier survivor function.

**Supplementary Figure 2. Histograms of the propensity score and the inverse-probability-of-treatment-weights (IPTW).**

**Supplementary Figure 3. IPTW-weighted Kaplan-Meier curves of overall survival according to treatment assignment to 2LCTX+BSC versus BSC alone.** For this analysis the simplified IPTW using an 8-variable propensity score model was used. *Number of patients represent the number in the synthetic pseudo-population generated by the IPTW. Abbreviations: IPTW – Inverse probability of treatment weight, 2LCTX – 2^nd^-line chemotherapy, BSC – best supportive care.

**Supplementary Figure 4. Landmark analysis of survival outcomes in patients who did and did not receive 2LCTX within 28 days.** Twenty-eight patients received 2LCTX within the first 28 days after progression. The long-dashed blue line represents the landmark date at 28 days. Abbreviations: 2LCTX – 2^nd^-line chemotherapy, BSC – best supportive care.
